# Synthesis of a Novel Water-Soluble Polymer Complexant Phosphorylated Chitosan for Rare Earth Complexation

**DOI:** 10.3390/polym14030419

**Published:** 2022-01-21

**Authors:** Yuxin Chen, Yujuan Chen, Dandan Lu, Yunren Qiu

**Affiliations:** School of Chemistry and Chemical Engineering, Central South University, Changsha 410083, China; yxchen1230@163.com (Y.C.); xk1257713404@163.com (Y.C.); ludandan0707@163.com (D.L.)

**Keywords:** polymer complexant, rare earth separation, phosphorylated chitosan, complexation–ultrafiltration

## Abstract

Combining the characteristics of rare earth extractants and water-soluble polymer complexants, a novel complexant phosphorylated chitosan (PCS) was synthesized by Kabachnik–Fields reaction with alkalized chitosan, dimethyl phosphonate, and formaldehyde as raw materials and toluene-4-sulfonic acid monohydrate (TsOH) as catalyst. The complexation properties of PCS and poly (acrylic acid) sodium (PAAS) for lanthanum ions in the solution were compared at the same pH and room temperature. In addition, the frontier molecular orbital energies of polymer–La complexes were calculated by the density functional theory method, which confirmed the complexation properties of the polymers to rare earths. The results indicate that the PCS has better water solubility compared with chitosan and good complex ability to rare earths, which can be used for rare earth separation by the complexation–ultrafiltration process.

## 1. Introduction

Rare earth elements, known as industrial vitamins, are wildly used in military industry, electronics, chemical industry, metallurgy, and other fields [1]. The efficient separation of rare earths is the key to the development of the rare earth industry. At present, solvent extraction is the main method for the rare earth separation in the industry [2,3]. Although a series of novel extractants containing multiple different coordination functional groups have been developed, such as α-aminophosphonate extractant [4,5], to improve the efficiency of concentration and separation, inevitable disadvantages still exist, such as low separation efficiency of single stage, large amount of consumption of acid and alkali, and the loss of extractants [6,7,8].

Complexation–ultrafiltration (C–UF) uses water soluble polymer complexants, of which the molecular weight is greater than the molecular weight cut-off (MWCO) of the ultrafiltration membrane. In addition, the complexants have an amount of nitrogen, sulfur, phosphorus or carboxyl groups in order that they can bond with the metal ions. Moreover, the complexed ions can be rejected by the ultrafiltration membrane, while the free ions can permeate the UF membrane [9,10,11,12]. C–UF has been widely used for the concentration and separation of heavy metal ions, and the used complexants are poly (acrylic acid) sodium (PAAS), copolymer of maleic acid and acrylic acid (PMA), chitosan (CS), etc. [13,14]. However, there are few reports regarding the separation of rare earths by C–UF. In addition, after primary exploration, it was found that the rare earth separation using PAAS and PMA as complexants was not satisfactory due to the poor complexation performance of carboxyl groups to rare earths. Therefore, the design and synthesis of novel complexants for efficient separation of rare earths is of great significance, according to the characteristics of rare earth extractants and the polymer complexants.

Chitosan is a type of non-toxic and harmless biopolymer obtained from the natural polymer chitin. It is used in the field of water treatment due to its richness in amino and hydroxyl groups and good adsorption capacity to metals [15]. However, chitosan has some defects, such as low adsorption selectivity for metals and poor solubility, which limit its application. Therefore, a modified chitosan with excellent performance is often obtained by functional group modification to expand the application range of chitosan [16]. Although there are a large number of hydroxyl groups in the structure of chitosan, they can provide fewer modified active sites due to the steric hindrance effect and the charge carried by the groups, while most of the amino groups in chitosan are in a free state and their reaction activity is significantly greater than hydroxyl sites [17].

Herein, combining the structural characteristics of rare earth extractants and chitosan, we proposed the grafting of phosphoryl groups onto amino groups of chitosan to obtain α-substituted phosphorylated chitosan (PCS), which can be used as a novel complexant for rare earth concentration and separation by C–UF. The PCS was characterized by Fourier transform infrared (FTIR), X-ray diffraction (XRD), and scanning electron microscopy (SEM), etc. The results show that the PCS has better water solubility compared with chitosan and good complex ability to rare earths. Moreover, it has a good application prospect in the field of rare earth separation.

## 2. Materials and Methods

### 2.1. Materials and Membrane

Chitosan with the average molecular weight of 300 kDa and deacetylation degree of 80% was purchased from Shanghai Macklin Biochemical Co., Ltd. (Shanghai, China). Poly (acrylic acid) sodium (PAAS, MW = 250 kDa) was provided by Tianjin Guangfu Fine Chemical Research Institute (Tianjin, China). Dimethyl phosphonate was obtained from Shanghai Yi En Chemical Technology Co., Ltd. (Shanghai, China). Toluene-4-sulfonic acid monohydrate (TsOH) and sodium hydroxide were supplied by Sinopharm Chemical Reagent Co., Ltd., Shanghai, China. Lanthanum oxide (La_2_O_3_), dehydrated alcohol, acetone, acetic acid, and 37~40% of formaldehyde solution (all provided by Chengdu Chron Chemicals Co., Ltd., Chengdu, China) were used. MD44 dialysis bags (average diameter of 28 mm) with the retention range from 8 to 14 kDa were bought from Shanghai Leibusi Company (Shanghai, China). All of the chemical reagents in this experiment were analytical pure.

### 2.2. Methods

#### 2.2.1. Synthesis of the PCS

To weaken the intermolecular force of chitosan, 10 g of chitosan was added into a 250 mL beaker containing 50 g of sodium hydroxide and 150 mL of ultrapure water under stirring at room temperature. After mixing evenly, it was placed in the refrigerator for 8 days. The alkalized chitosan was obtained after thawing and removing the alkaline solution by suction filtration. 

Thereafter, 1 g of alkalized chitosan dissolved in 150 mL of 1% acetic acid solution and 4 mL of dimethyl phosphonate were mixed in a three-port flask under stirring. Then, the temperature was raised to 65 °C. In addition, 10 mL of formaldehyde solution (37~40%) and 0.1 g of TsOH as reaction catalyst were added to the system. After refluxing for 5 h, the reaction solution was transferred to a dialysis bag at room temperature for 48 h for the removal of low molecular weight soluble impurities to obtain the high purity product. After vacuum distillation, the mixture of absolute ethanol (200 mL) and acetone (200 mL) was added to the concentrated solution to precipitate. Then, after filtering, the precipitates were washed with absolute ethanol and acetone for several times, and vacuum dried at 50 °C to obtain the pure polymer phosphorylated chitosan (PCS).

#### 2.2.2. Procedure of C–UF

The ultrafiltration membrane device used in this study was the polyvinyl butyral (PVB) hollow fiber membrane with molecular weight cut-off (MWCO) of 20 kDa [18]. Initially, the complexants were pretreated using diafiltration to remove the small polymers. Then, the complex solution of polymer and rare earths at different P/RE values (mass ratio of complexant to rare earth ions) was prepared. After contacting for about 2 h, the obtained materials were injected into the hollow fiber membrane module by a peristaltic pump at the feed rate of 30 L h^−1^. The permeates were collected for 30 s after 5 min and further analyzed to determine the rare earth ions and polymer concentration. All of the experiments were performed at 25 °C.

### 2.3. Characterization

The functional groups between the raw materials and products were characterized by Fourier transform infrared (NicoletiS50, Thermo Fisher Scientific, Waltham, MA, USA). The synthesized PCS and CS were dissolved in deuterium oxide (D_2_O) to prepare NMR samples, and the ^1^H NMR of samples was detected with a 500 MHz BRUKER spectrometer. The crystallinity of chitosan and PCS was tested by X-ray diffraction (Advance D8). The morphology of the products was observed by SEM on a JSM-7610F field emission scanning electron microscope.

## 3. Results and Discussion

**Scheme 1:**
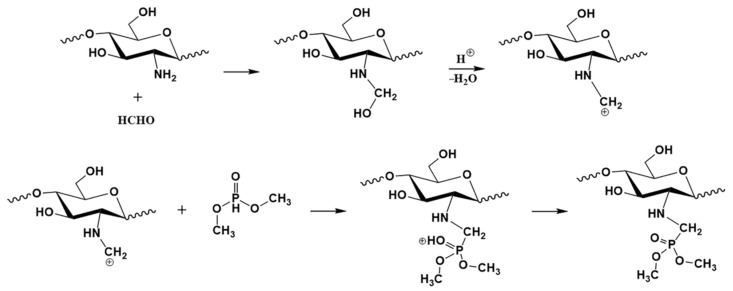
Synthesis mechanism for the PCS.

Scheme 1 shows the synthesis mechanism for the PCS. The amino group on chitosan was condensed with formaldehyde under the catalysis to obtain methylene-amine positive ions, and then continued to undergo an electrophilic substitution reaction with the active hydrogen of dimethyl phosphonate to obtain the product. The solubility of PCS is higher than 2 mg mL^−1^ in water, which is significantly higher than chitosan. 

**Figure 1:**
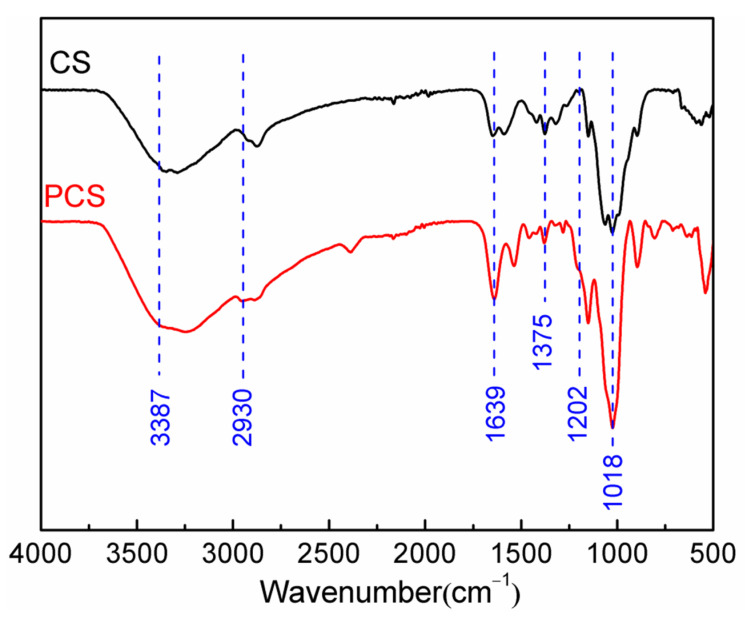
FTIR spectra of the PCS and chitosan.

Figure 1 shows the Fourier transform infrared (FTIR) absorption spectra of the PCS and chitosan. The chitosan powder exhibits characteristic absorption bands of methylene group antisymmetric stretching vibration at 2930 cm^−1^, C–N stretching vibration (coupled with N–H bending) at 1375 cm^−1^, N–H bending (coupled with C–N stretching) vibration of amide groups at 1639 and 1592 cm^−1^, and C–O stretching vibration at 1061 cm^−1^. The broad band observed at 3200–3500 cm^−1^ is attributed to the intermolecular and intramolecular hydrogen bonding of –NH_2_ and –OH stretching vibration of chitosan, which is O–H stretching at 3440 cm^−1^, as well as N–H asymmetric stretching at 3387 and 3284 cm^−1^ [19]. In the case of PCS, characteristic bands of P=O stretching vibration at 1202 cm^−1^ and P–O–C antisymmetric stretching vibration at 1018 cm^−1^ [20] were shown, which illustrate that the phosphoryl groups are successfully grafted onto the chitosan structure. In addition, the bond at 1375 cm^−1^ (C–N–H bending) of PCS is clearly smaller than the chitosan, suggesting that the grafting reaction mainly occurs on the amino groups.

**Figure 2:**
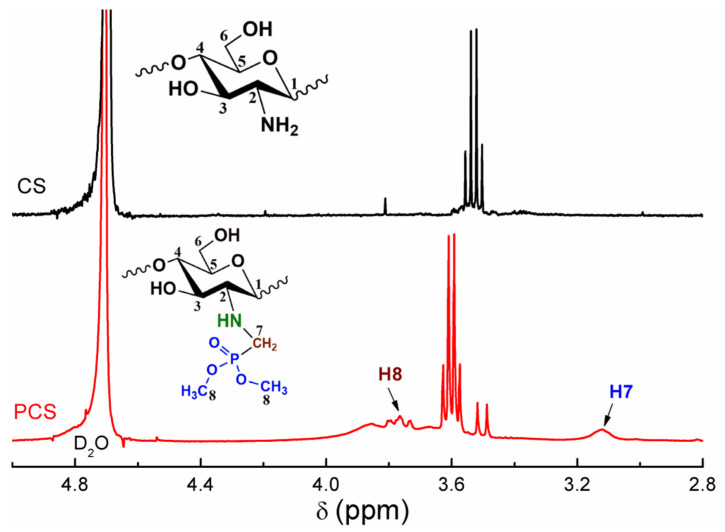
^1^H NMR spectra of the PCS and CS.

The ^1^H NMR spectra of the unmodified chitosan and PCS are shown in Figure 2. In the spectrum of chitosan, the signals in the range of 3.36–3.81 ppm are the resonance of H2–H6 protons in the heterocycle. In the case of PCS, the chemical shift at 3.11 ppm represents the proton signal of methylene grafted to the amino position. In addition, the emerging signal at 3.76 ppm is ascribed to the methyl group on the phosphoryl group, which makes it clear that the phosphoryl group is only grafted on the methylene.

**Figure 3:**
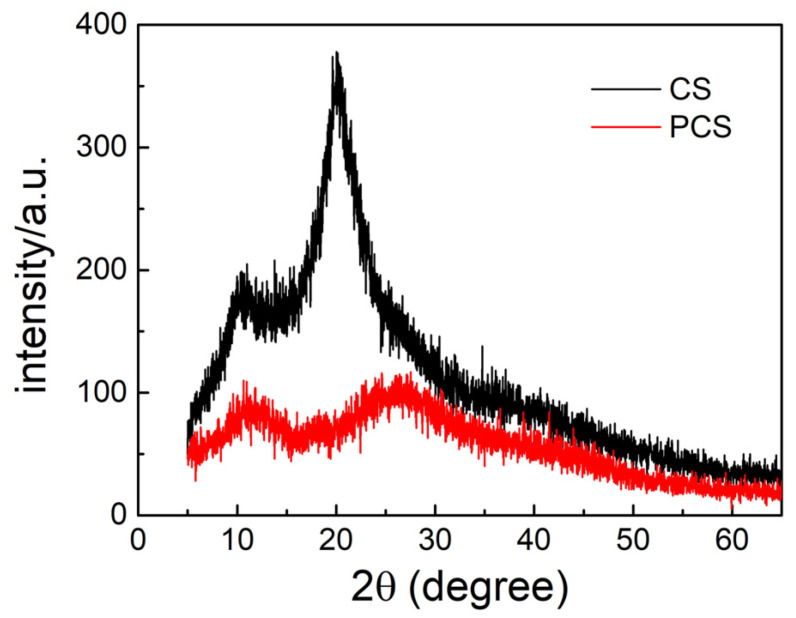
X-ray diffraction (XRD) patterns of the PCS and CS.

The crystallinity of chitosan and PCS is compared by X-ray diffraction (XRD) analysis, as shown in Figure 3. Intramolecular and intermolecular hydrogen bonds were found in the structure of chitosan. Therefore, chitosan shows strong crystallinity and poor solubility [21]. In addition, chitosan has a main crystalline peak at 2θ = 20° and an amorphous peak around 2θ = 10°. After modification, both peaks of chitosan are significantly reduced and tend to be flat. As a result, the grafted substituents destroyed the original crystalline structure of chitosan, mainly the hydrogen bond at the amino position, and the molecule almost lost its crystallization ability. This is the main reason for the good water solubility of the PCS. 

**Figure 4:**
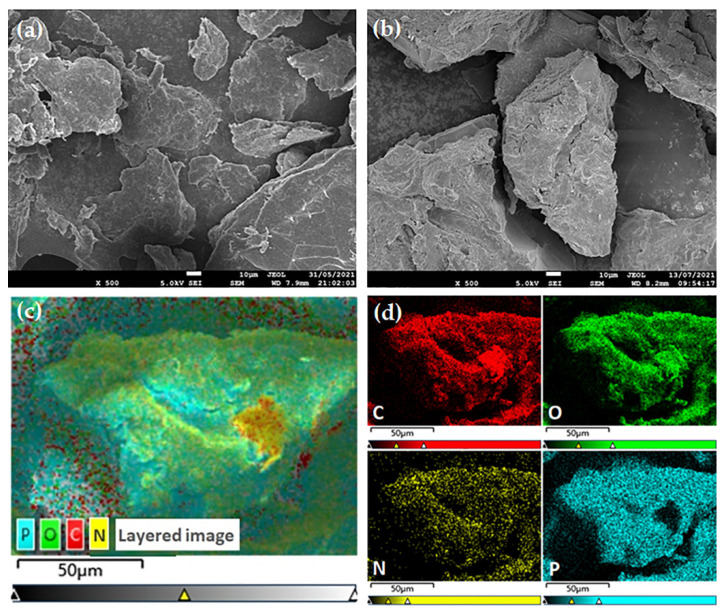
SEM images of chitosan (**a**) and the PCS (**b**). SEM–EDS mapping images of the PCS (**c**,**d**).

Figure 4a,b shows SEM images of raw materials of chitosan and the PCS at the same magnification, respectively. Compared with chitosan, the surface of PCS is rough and porous, and its cross section shows a porous morphology, which is related to the good water solubility of the PCS. The element distribution of PCS is analyzed by SEM–EDS mapping (Figure 4c,d). The results show that phosphorus entered the chitosan structure, and the C, O, N, and P elements of PCS are evenly distributed.

**Figure 5:**
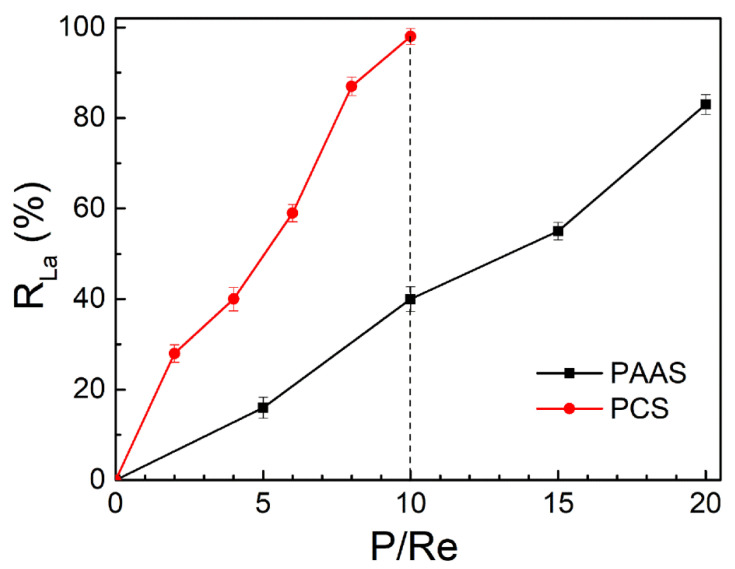
Comparison of complexation ability of the PCS and PAAS to La^3+^ at pH = 7.0.

In addition, the complexation ability to rare earth ions of PCS was studied. The ultrafiltration membrane was used to reject the complex formed by the complexants and rare earths, and the rejection effect was expressed by the rejection rate (R). The complexation ability of PCS and PAAS to lanthanum ions (La^3+^) at pH = 7.0 is shown in Figure 5. The data are the average of three experimental results. The PCS can basically complexate 98% of La^3+^ when P/Re is 10, while PAAS can only complexate 82% of lanthanum ions in the solution when P/Re is 20, which indicates that the PCS has better complexation ability to rare earths than PAAS.

**Figure 6:**
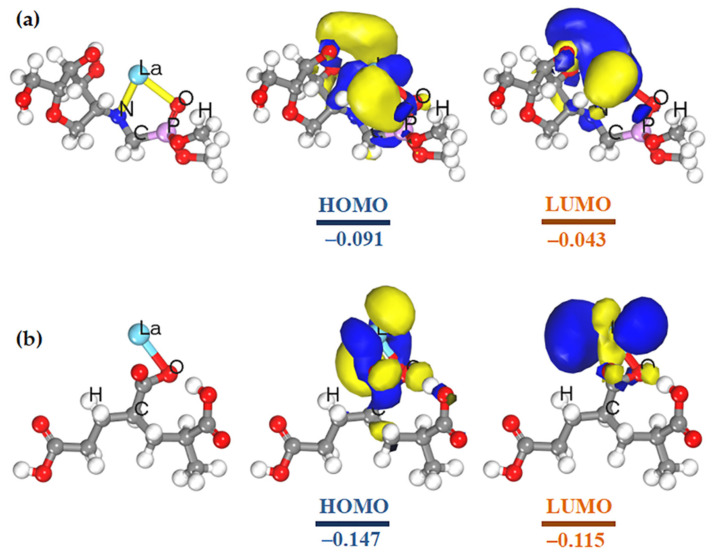
Isosurfaces and energy of the stable configuration (**left**), HOMOs (**middle**), and LUMOs (**right**) of (**a**) PCS–La, (**b**) PAA–La complexes.

By analyzing the molecular structure of rare earth extractants, it is concluded that organophosphorus extractants are generally coordinated with metal ions through the P=O group, while amine extractants are coordinated with metal ions through nitrogen atoms, and the extraction order of rare earths by these two extractants is opposite [22,23,24]. The α-aminophosphonate compounds containing both phosphoryl and amino groups have two coordination atoms, which can form a more stable structure with rare earths in a wide range of acidity, and have better complexation and separation properties to rare earths [25]. Therefore, it is not difficult to understand that the P=O and nitrogen atom of the PCS can coordinate with rare earths at the same time to form a stable five-membered ring structure, while PAAS only complexes with rare earths through carboxyl groups. This is the reason why the PCS shows good rare earth complexation ability. Moreover, the adsorption properties of PCS and PAAS with La(III) have been calculated with the DMol3 electronic structure code (Materials Studio 2019) founded on the density functional theory GGA/BLYP/DNP (Figure 6a,b). The energy gap (∆E = E_LUMO_ − E_HOMO_) represents the chemical activities of the molecule. The ∆E of PCS–La and PAA–La complexes is 0.047 and 0.032 au, respectively. This confirms that the complexation ability of the PCS to La is stronger than PAAS at the molecular level. 

## 4. Conclusions

In conclusion, phosphorylated chitosan (PCS) with good water solubility is successfully designed and synthesized. Benefiting from the amino and phosphoryl groups contained in the structure of PCS, the PCS shows excellent complexing performance to rare earth ions, which provides a good application prospect as an excellent rare earth complexant. Moreover, the green and convenient synthesis process represents a potential possibility for large-scale industrial production of the PCS in the future.

## Data Availability

Not applicable.

## References

[1] Balaram V. (2019). Rare earth elements: A review of applications, occurrence, exploration, analysis, recycling, and environmental impact. Geosci. Front..

[2] Costa T., Silva M., Vieira M. (2020). Recovery of rare-earth metals from aqueous solutions by bio/adsorption using non-conventional materials: A review with recent studies and promising approaches in column applications. J. Rare Earths.

[3] Xie F., Zhang T., Dreisinger D., Doyle F. (2014). A critical review on solvent extraction of rare earths from aqueous solutions. Miner. Eng..

[4] Lu Y., Zhang Z., Li Y., Liao W. (2017). Extraction and recovery of cerium(IV) and thorium(IV) from sulphate medium by an α-aminophosphonate extractant. J. Rare Earths.

[5] Zhao Q., Zhang Z., Li Y., Bian X., Liao W. (2018). Solvent extraction and separation of rare earths from chloride media using α-aminophosphonic acid extractant HEHAMP. Solvent Extr. Ion Exch..

[6] Jyothi R.K., Thenepalli T., Ji W., Parhi P.K., Lee J. (2020). Review of rare earth elements recovery from secondary resources for clean energy technologies: Grand opportunities to create wealth from waste. J. Clean. Prod..

[7] Hidayah N.N., Abidin S.Z. (2018). The evolution of mineral processing in extraction of rare earth elements using liquid-liquid extraction: A review. Miner. Eng..

[8] Xiao Y., Long Z., Huang X., Feng Z., Cui D., Wang L. (2013). Study on non-saponification extraction process for rare earth separation. J. Rare Earths.

[9] Desai K.R., Murthy Z.V.P. (2012). Removal of silver from aqueous solutions by complexation–ultrafiltration using anionic polyacrylamide. Chem. Eng. J..

[10] Barakat M.A., Schmidt E. (2010). Polymer-enhanced ultrafiltration process for heavy metals removal from industrial wastewater. Desalination.

[11] Tang S., Qiu Y. (2018). Removal of copper(II) ions from aqueous solutions by complexation–ultrafiltration using rotating disk membrane and the shear stability of PAA–Cu complex. Chem. Eng. Res. Des..

[12] Gao J., Qiu Y., Ben H., Zhang Q., Zhang X. (2018). Treatment of wastewater containing nickel by complexation- ultrafiltration using sodium polyacrylate and the stability of PAA-Ni complex in the shear field. Chem. Eng. J..

[13] Tang S., Qiu Y. (2019). Selective separation and recovery of heavy metals from electroplating effluent using shear-induced dissociation coupling with ultrafiltration. Chemosphere.

[14] Zhou H., Qiu Y., Le H. (2019). Recovery of metals and complexant in wastewater by shear induced dissociation coupling with ultrafiltration. J. Appl. Polym. Sci..

[15] Zemmouri H., Drouiche M., Sayeh A., Lounici H., Mameri N. (2012). Coagulation Flocculation Test of Keddara’s Water Dam Using Chitosan and Sulfate Aluminium. Procedia Eng..

[16] Wang J., Wang L., Yu H., Abdin Z., Chen Y., Chen Q., Zhou W., Zhang H., Chen X. (2016). Recent progress on synthesis, property and application of modified chitosan: An overview. Int. J. Biol. Macromol..

[17] Izumrudov V., Volkova I., Grigoryan E., Gorshkova M. (2011). Water-soluble nonstoichiometric polyelectrolyte complexes of modified chitosan. Polym. Sci..

[18] Xu J., Tang S., Qiu Y. (2019). Pretreatment of poly (acrylic acid) sodium by continuous diafiltration and time revolution of filtration potential. J. Cent. South Univ..

[19] Liu X., Zhang L. (2015). Removal of phosphate anions using the modified chitosan beads: Adsorption kinetic, isotherm and mechanism studies. Powder Technol..

[20] Zhao D., Xu J., Wang L., Du J., Dong K., Wang C., Liu X. (2019). Study of two chitosan derivatives phosphorylated at hydroxyl or amino groups for application as flocculants. J. Appl. Polym. Sci..

[21] Yang T., Huang N., Meng L. (2013). Chitosan modified by nitrogen-containing heterocycle and its excellent performance for anhydrous proton conduction. RSC Adv..

[22] Lu Y., Wei H., Zhang Z., Li Y., Liao W. (2016). Selective extraction and separation of thorium from rare earths by a phosphorodiamidate extractant. Hydrometallurgy.

[23] Wei H., Li Y., Zhang Z., Xue T., Kuang S., Liao W. (2017). Selective Extraction and Separation of Ce (IV) and Th (IV) from RE(III) in Sulfate Medium using Di(2-ethylhexyl)-N-heptylaminomethylphosphonate. Solvent Extr. Ion Exch..

[24] Liao W., Zhang Z., Kuang S., Wu G., Li Y. (2017). Selective extraction and separation of Ce(IV) from thorium and trivalent rare earths in sulfate medium by an α-aminophosphonate extractant. Hydrometallurgy.

[25] Zhao Q., Li Y., Kuang S., Zhang Z., Bian X., Liao W. (2019). Synergistic extraction of heavy rare earths by mixture of α-aminophosphonic acid HEHAMP and HEHEHP. J. Rare Earths.

